# Assessment of Occupational Exposure to Inhalable Aerosols in an Instant Powdered Food Manufacturing Plant in Norway

**DOI:** 10.1016/j.shaw.2024.05.001

**Published:** 2024-05-15

**Authors:** Christine Darbakk, Pål Graff, Raymond Olsen

**Affiliations:** 1 National Institute of Occupational Health (STAMI), Oslo, Norway; 2 University of Oslo, Oslo, Norway

**Keywords:** Food industry, Inhalable aerosols, Occupational exposure, Personal sampling, Powders

## Abstract

**Background:**

In the food manufacturing industry, exposure to inhalable aerosols contributes to respiratory illnesses such as occupational asthma and rhinitis. However, there is a lack of comprehensive exposure assessment studies. This study evaluated occupational exposure to inhalable aerosols in an instant powdered food manufacturing plant during work operations involving dried food and powders.

**Methods:**

In total, 50 workers from an instant powdered food manufacturing plant were recruited. Personal inhalable aerosol exposure measurements were taken for both full-shift and task-based activities. The concentrations of inhalable aerosols were analyzed to identify any variation within and across departments, as well as between seasons, handedness, and sex.

**Results:**

In total, 134 personal air samples were collected, and the particulate mass was determined gravimetrically. The concentrations of inhalable aerosols ranged from 0.1 to 27 mg/m^3^ for full-shift exposure measurements and 3.1 to 73 mg/m^3^ for task-based measurements. Statistically significant differences in mean aerosol concentrations were found across departments (A:B *p* < 0.001, A:C *p* < 0.05, B:C *p* < 0.001) and between seasons (*p* < 0.001).

**Conclusion:**

This study revealed high exposure to inhalable aerosols among workers, particularly those involved in manual weighing, mixing, and adding powders. The significant differences between departments highlight the specific activities contributing to increased inhalable aerosol concentrations. Seasonal variations were also evident, with autumn showing higher concentrations of inhalable aerosols in all departments compared with summer. These findings emphasize the importance of understanding the distribution of aerosol concentrations across different work tasks and departments, particularly during different seasons.

## 1 Introduction

The food industry is a large and diverse sector where millions of workers are exposed to aerosols in wet and dry working environments. For example, those working in the seafood industry may encounter aerosols in wet conditions, while those in bakeries may encounter them in dry conditions [1,2]. These aerosols contain various components, including food allergens and endotoxins, and have been linked to various adverse respiratory health outcomes such as allergies, infectious diseases, acute toxic reactions, hypersensitivity pneumonitis, and even cancer development [[3], [4], [5], [6], [7]]. Therefore, occupational exposure in the food manufacturing industry presents a substantial occupational health challenge [4].

Recent epidemiological studies have examined work-related symptoms, such as rhinitis and asthma, among plant food handling employees. These studies have primarily focused on individuals working in flour mills, bakeries, and the seafood industry [1,2,[8], [9], [10]]. It has been found that rhinitis is 2 to 4 times more common than occupational asthma [11], and 10% to 25% of occupational asthma and rhinitis cases can be attributed to inhaling food-related substances during workplace food handling and processing activities. The prevalence of occupational asthma varies across different industries, with rates of 3%–10% in individuals working with green coffee beans, 4%–13% in bakers, 2%–8% in those processing bony fish, and 4%–36% in shellfish processing workers [[12], [13], [14]].

Recent research has identified new seafood allergens, such as Pen m 4 and creatine kinase, in workers exposed to dry shrimp Gammarus powder and fish [15,16]. Furthermore, peach tree pollen has been linked to allergic sensitization in farm workers in peach orchards because it contains the allergen Primus persica 9 [17]. Inhalation of quinoa flour has been linked to respiratory problems in mill workers [18]. Exposure to both liquid and powdered forms of lyophilized donkey milk has caused occupational allergies in food laboratory analysts [19]. In cheese production, substances such as rennet and locust bean gum have also been reported to trigger occupational rhinitis or asthma among cheese makers [[20], [21], [22]]. There have also been reported cases of respiratory issues among butchers involved in sausage production [23], and a case of occupational asthma related to saponins from soap nuts and Quillaja bark used in beer production [24].

Despite numerous studies on the immunological effects of occupational exposure, only a limited number have specifically studied aerosol exposure in the food manufacturing industry. Some clinical studies have linked inhalation of food products such as dried fruits, teas, spices, herbs, coffee, and castor with respiratory problems such as asthma and rhinitis [[25], [26], [27], [28], [29], [30], [31], [32]]. However, these studies mainly focused on documenting symptoms, conducting skin prick tests, and assessing lung function in workers. To the best of our knowledge, except for exposure assessments in bakeries and the seafood industry, there is a lack of research on aerosol measurements in other food production facilities.

The production of powdered foods plays a substantial role globally, allowing for preserving without compromising quality for extended periods. Various foods undergo mechanical processing to achieve a finely granulated form, including grain flour, cereal flour, dried egg powder, dried milk powder, dried spices and herbs, and fruit or vegetable powders. In addition, certain additives, such as enzymes, flavorings, colorants, sulfites, and thickening agents may be intentionally added to food products to enhance flavor, color, consistency, and shelf life [33]. These substances can become airborne in various industrial settings, such as bakeries, flour mills, food processing plants, dairy facilities, spice and flavoring production facilities, confectionery and snack factories, and nutritional supplement and health food production environments. The handling, processing, and packaging of powdery substances in these environments can generate airborne particles, potentially exposing workers to them.

Despite the widespread recognition of the impact of aerosol exposure on human health, there is a lack of occupational exposure limits specifically for food allergens. Moreover, the existing limits vary across different countries. For instance, the 8-hour time-weighted average for flour dust ranges from 0.5 mg/m^3^ in Belgium to 10 mg/m^3^ in the United Kingdom [34]. Except for a few substances, such as flour dust [35], there is limited documentation on the exposure-response relationships, and knowledge of threshold values within this industry is also lacking. Therefore, conducting more comprehensive occupational exposure assessments is crucial in expanding our understanding of the aerosol concentration levels in the food industry.

This study aimed to evaluate occupational aerosol concentrations and identify high-exposure work tasks among employees at a powdered food manufacturing facility in Norway.

## 2 Materials and methods

### 2.1 Study design

The aerosol exposure assessment was conducted through two sampling campaigns at a food manufacturing plant located in Norway. The first sampling campaign was conducted in June, covering 3 d and 5 shifts. The second sampling campaign was conducted in November, covering 2 d and 3 shifts. In total, 50 workers, consisting of 26 men and 24 women, voluntarily participated in the sampling campaigns. In collaboration with the factory management, workers in each department who were believed to have the highest exposure to powders were recruited. The work shift measurements were categorized into three groups based on the specific job tasks of the participants within the manufacturing plant. In Department A (*N* = 13), participants were responsible for producing powder nutrients through processes involving grinding and powder production. In Department B (*N* = 29), participants engaged in activities related to handling raw powder nutrients, including weighing, mixing, and filling powders into finished products. In Department C (*N* = 25), participants handled the final powder materials enclosed in big bags during the filling process for consumer-packaged products. Their responsibilities were also included manually folding and depositing empty big bags into a recycling shaft and operating machinery for packaging final consumer products. Overall, the tasks performed by the workers were categorized as having a moderate workload. Heavy lifting was facilitated using forklifts, cranes, and assistive devices, while bags weighing less than 10 kg were lifted manually.

During their 8-h work shifts, each participant carried air sampling equipment and completed a questionnaire that gathered personal details, such as their name, sex, birth year, handedness, occupation, and work-related information, including the tasks performed during sampling and using respiratory protection equipment. The study included 50 participants, resulting in 67 parallel measurements. Of these, 35 participants were measured for 1 d, 13 for 2 d, and 2 for 3 d making 134 samples. Among these, 65 measurements were taken for a full shift, with a minimum of 310 min, a maximum of 445 min, and a mean of 380 min. In addition, two measurements were task-based with a sampling time of less than 120 min.

The plant manufactured powdered food products, such as broth, soup, sauce, stews, pasta dishes, beverages, waffles, pancake and cake mixes, pizza mixes, and rice. The production process primarily involved a semi-automatic big bag system, where raw materials were ground in mills and loaded into big bags automatically. Workers were responsible for manual tasks such as positioning, opening, and transporting big bags using forklift trucks while filling and unloading powdered products. They also manually weighed and added ingredients and additives to the big bags.

The plant has a mechanical ventilation system with air filtration in each department. The operation of the ventilation system varies depending on the department and the season. In the winter, the system runs continuously from Monday to Friday in certain areas and every day from 6 AM to 12 AM in other areas, depending on the production processes. However, in the summer, the ventilation system operates continuously in all departments to maintain a consistent temperature and humidity level throughout the factory and its departments.

### 2.2 Sampling procedure

#### 2.2.1 Personal sampling

Inhalable aerosol was collected on 37 mm polyvinyl chloride (PVC) filters with a pore size of 5 μm (Merck Millipore, Burlington, Massachusetts, USA). The filters were contained in a GSP inhalable aerosol sampler (GSA Messgerätebau, Ratingen, Germany), which was connected to a Casella APEX 2 Pro (Casella UK, Bedford, UK) air sampling pump and operated at an air flow rate of 3.5 L/min. Each participant carried a backpack equipped with two GSP samplers, one on each side of the backpack straps, within their breathing zone. The air sampling flow rates were measured before and after sampling using a Bios Defender 510-M primary air flow meter (Bios International Corp., New York, USA).

#### 2.2.2 Stationary sampling

For stationary sampling, a direct reading dust sampler (DustTrak DRX aerosol monitor TSI, model 8533, MN, USA) was used at two different departments for an entire morning shift and an entire day (morning and evening shift). The sampler logged aerosol concentrations every 10 s. The total dust was calibrated against an external 25-mm PVC filter with a pore size of 5 μm, contained in antistatic polypropylene filter cassettes (Teknolab AS, Norway). The external filter cassette was attached to a Casella APEX 2 Pro (Casella UK, Bedford, UK) with an air flow rate of 2.0 L/min.

#### 2.2.3 Analytical method

The collected particulate mass was determined gravimetrically after the filters were conditioned for at least 24 h in a climate-controlled room (20°C ± 1°C and relative humidity of 40% ± 2%) before and after sampling. A Sartorius MSA 6.6S microbalance (Sartorius AG, Göttingen, Germany) was used for the analysis. The filters were discharged before weighing using a Polonium-210 α-emitter (StaticMaster™, Nuclear Products Co, CA, USA). The limits of detection (LOD) were defined as three times the standard deviation of 6 unexposed filters and found to be 0.024 mg/filter for the GSP sampler and 0.010 mg/filter for the total dust filter cassette. The aerosol concentrations were then calculated based on the sampled air volumes. None of the exposed filters were below the LOD.

### 2.3 Statistical analysis

Statistical analyses were performed using the R/RStudio version 4.2.3 [36], with the rstatix, lme4, and lmerTest packages [[37], [38], [39]]. Graphical presentations were created using the ggplot2 package [40].

The data were assessed for normality using the Shapiro–Wilk test [41], with a *p*-value of less than 0.05 considered statistically significant. Because the data did not follow a normal distribution, log transformation was applied for subsequent analyses. In graphical visualizations, *p*-values were denoted using asterisks: a single asterisk (∗) indicated *p*-values less than 0.05, double asterisks (∗∗) indicated *p*-values less than 0.01, and triple asterisks (∗∗∗) indicated *p*-values less than 0.001.

The Welch's *t*-test [42] was utilized to determine any discrepancies in inhalable aerosol concentrations among departments and seasons for full-shift measurements. For visual representations of nontransformed data, the nonparametric Wilcoxon signed-rank test was employed [43]. The correlation between full-shift measurements on the right and left sides was examined using Pearson correlation [44]. A Bland–Altman plot was utilized to assess variations between measured inhalable aerosol concentrations with the sampler placed on the left or right side [45]. Furthermore, a linear mixed-effect model (LMER) was used to evaluate the impact of handedness, sex, shift, department, and season, as well as the interaction between season and department, on the inhalable aerosol concentrations during a full shift. The participant's ID was included as a random effect to account for potential discrepancies among participants.

Log concentration (mg/m^3^) ∼ Handedness + Sex + Shift + Department + Season + Season: Department + (1|ID)

where handedness is categorized as right or left, sex as male or female, shift as morning or evening, department as A, B or C, season as summer or autumn, and ID as p01-p49.

## 3 Results

### 3.1 Personal exposure measurements

In total, 134 samples were collected from participants, with two samples collected per person. However, seven of these samples had to be discarded due to errors in the sampling process. Of the remaining 127 samples, 123 were taken for a full work shift (62 from the right-sided GSPs and 61 from the left-sided GSPs), while the remaining four were taken during specific tasks (two from the right-sided GSPs and two from the left-sided GSPs). The task-based measurements showed concentrations of 3.4 and 63 mg/m^3^ on the right side and 3.1 and 73 mg/m^3^on the left side.

The results of the full-shift measurements are shown in Fig. 1. The median for the inhalable aerosol measurements during the full work shift was 1.1 mg/m^3^ for the GSPs attached to the right side of the chest (represented by the black horizontal line in the gray violin plot in Fig. 1). The aerosol concentrations ranged from 0.1 to 20 mg/m^3^. For the GSPs attached to the left side, the median was 1.4 mg/m^3^ (shown by the black horizontal line in the orange violin plot in Fig. 1), with aerosol concentrations varying from 0.1 to 27 mg/m^3^. In 90% of the collected samples, the inhalable aerosol concentrations were below 10 mg/m^3^ and 9.2 mg/m^3^ for the right- and left-sided GSPs, respectively (as shown in Table 1).

**Fig. 1:**
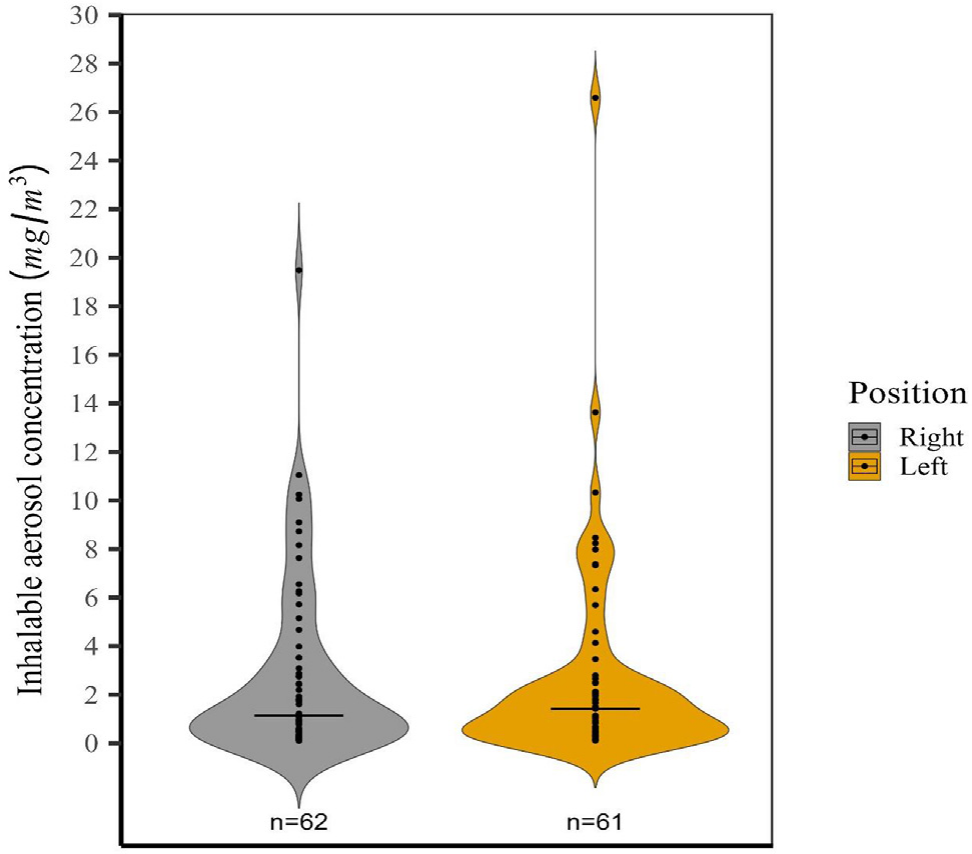
Personal sampling of inhalable aerosol concentration (mg/m^3^) for right (gray violin) and left (orange) full-shift measurements. The median, indicated by the black horizontal line, was 1.1 mg/m^3^ on the right side and 1.4 mg/m^3^ on the left side, with the width of the violin shape illustrating the frequency of the data at various values. According to the Wilcoxon signed-rank test, no statistically significant difference was observed in inhalable aerosol concentration between the GSP samplers (*p* = 0.74).

**Table 1:** Personal air sampling results for inhalable aerosol concentration in Department A:C. The results of the Wilcoxon signed-rank test show that there was no statistically significant difference in the concentration of inhalable aerosols between the right and left side GSP samplers in Departments A, B, and C. The *p-*values for these departments were 0.608, 0.610, and 0.971, respectively

	GSP (right side)			GSP (left side)		
Department	A	B	C	A	B	C
*N* =	13	27	22	13	27	21
AM (SD) (mg/m^3^)	1.3 (1.7)	4.7 (4.5)	1.3 (2.1)	1.2 (1.7)	4.6 (5.6)	1.2 (1.5)
Median (mg/m^3^)	0.6	3.1	0.5	0.5	2.5	0.5
90th percentile	4.1	10	2.8	2.0	9.2	2.5

LOD = 0.02 mg/m^3^ at a sampling time of 380 min and an airflow rate of 3.5 L/min.

In total, 62 and 61 samples were collected from the right and left-sided GSP sampler in Department A, B, and C, respectively (see Table 1). The mean and median concentrations of samples on the right side were consistently higher than those from the left in all three departments. However, these differences were not statistically significant (Department A: *p* = 0.608, Department B: *p* = 0.610, Department C: *p* = 0.971).

Fig. 2A depicts the relationship between inhalable aerosol concentrations collected by the two parallel GSP personal aerosol samplers. The graph demonstrates a strong positive correlation between the right GSP aerosol sampler and the left GSP aerosol sampler, with a correlation coefficient (r) of 0.92. The standard error is indicated by the gray shaded area, and the regression equation is *y* = −0.38 + 1.1x. Notably, there was no notable variation in inhalable aerosol concentrations between the left and right GSP aerosol samplers (Fig. 2B). Furthermore, when examining participants categorized by handedness, no consistent differences were observed in inhalable aerosol concentrations between the two parallel GSP samplers.

**Fig. 2:**
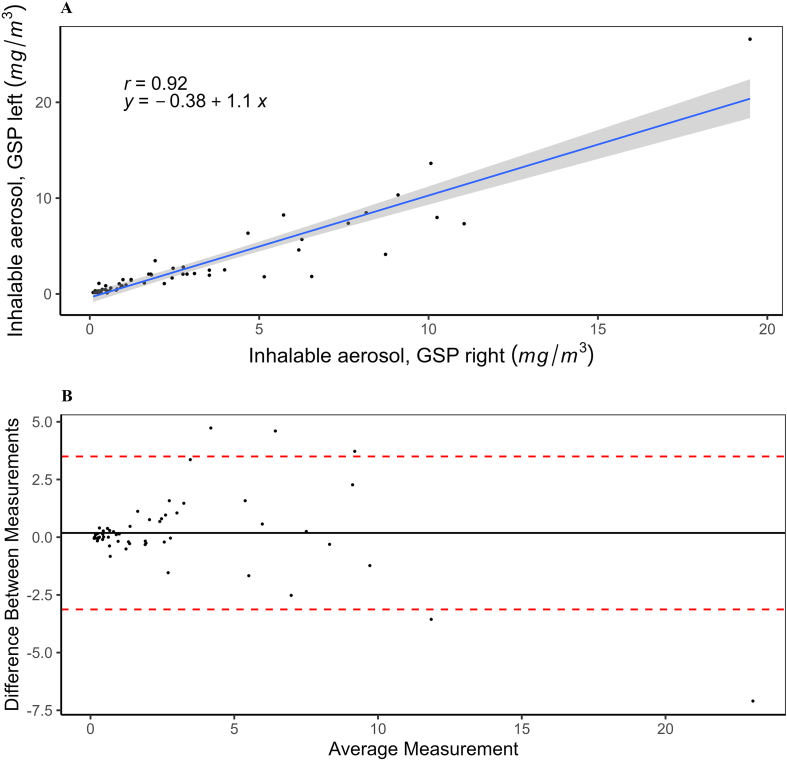
Scatterplot illustrating the correlation between the right and left GSP personal aerosol sampler, including a fitted regression line (blue line), the shaded gray area representing the standard error, correlation coefficient r = 0.92, and regression equation *y* = –0.38 + 1.1x (A). The differences in inhalable aerosol concentrations (mg/m^3^) between the right and left GSP are plotted against the mean of the two measurements, with the limits of agreement (red dotted line) depicted from –1.96s to +1.96s (B).

**Fig. 3:**
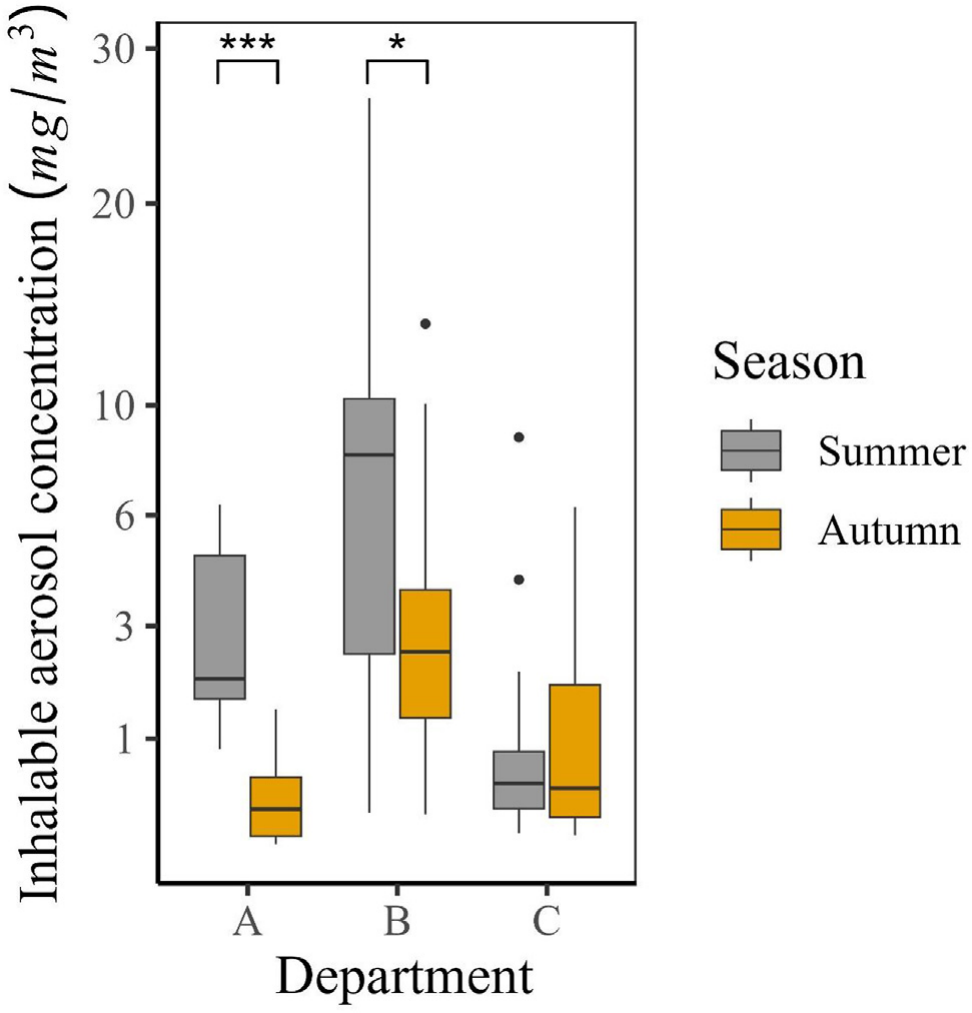
Seasonal variation in inhalable aerosol concentration (mg/m^3^) in personal air samples between Departments A: C. The *x*-axis represents the departments, while the *y*-axis shows the inhalable aerosol concentrations with a square root transformation. The black horizontal line in each box indicates the median concentration, and the black dots represent potential outliers. Statistical significance between seasons within departments were determined using the nonparametric Wilcoxon signed-rank test. An asterisk (∗) indicates a *p*-value less than 0.05, and triple asterisks (∗∗∗) indicate a *p*-value less than 0.001.

The study revealed that during autumn, the concentrations of inhalable aerosols were consistently higher in all departments compared with the summer season, as depicted in Fig. 3. Statistical analysis was conducted on log-transformed aerosol concentrations using Welch's *t*-test, which confirmed a statistically significant seasonal variation in Department A and B with *p*-values of < 0.001 and < 0.05, respectively. However, no statistically significant variation was observed in Department C. In addition, the analysis showed a difference in the variability of aerosol concentrations across departments. Department A displayed a noticeable difference between seasons, whereas Departments B and C showed similar levels of variability. Department B had the highest aerosol concentrations in the summer, followed by Departments C and A. Further, Welch's *t*-test indicated statistically significant differences in the mean aerosol concentrations among Department A and B, A and C, and B and C, with *p*-values of < 0.001, < 0.05, and < 0.001, respectively. During the autumn season, Department B continued to have the highest concentrations of aerosol, but Department A had higher concentrations than Department C. Statistically significant differences were observed in aerosol concentrations between Department A and C (*p* < 0.05) and B and C (*p* < 0.001), whereas no statistically significant difference was found between Departments A and B (A:B *p* = 0.146).

### 3.2 Linear mixed effect model of inhalable aerosol concentrations

The results of the linear mixed-effect model (LMER) analysis are presented in Table 2. The model was adjusted for handedness and sex. The analysis revealed a statistically significant difference in aerosol concentration between summer and autumn (*p* < 0.001). While there was no statistically significant difference in aerosol concentrations between Department A and Department B during summer (*p* = 0.383), a statistically significant difference was observed in Department C compared with Department A (*p* = 0.004). The impact of seasonal changes on inhalable aerosol concentrations were considerably more pronounced in Department A than in Departments B (*p* = 0.005) and C (*p* < 0.001). The analysis also highlighted considerable inter-participant variability (with an intraclass correlation coefficient, ICC, of 0.75). Fixed effects accounted for 40.3% of the variance in aerosol concentrations Marginal *R*^2^, while the full model, including both fixed and random effects, explained 82.3% of the variance (Conditional *R*^2^).

**Table 2:** Linear mixed-effect model output for the effect of log transformed inhalable aerosol concentrations. Statistically significant differences between Department A (reference) during the summer and other departments, as well as the variations in aerosol concentrations between seasons, are presented in bold to indicate their statistical significance (*p* < 0.05).

Predictors	Model output	
	Estimates	*p*
Season [Summer vs Autumn]	3.034	<0.001
Department [A vs B for Summer]	0.498	0.383
Department [A vs C for Summer]	1.605	0.004
Season [Summer vs Autumn] × Department [A vs B]	−2.099	0.005
Season [Summer vs Autumn] × Department [A vs C]	−2.963	<0.001
**Random effects**		
σ^2^	0.34	
τ_00__ID_	1.04	
ICC	0.75	
N _ID_	49	
Observations	123	
Marginal *R*^2^/Conditional *R*^2^	0.403/0.852	

### 3.3 Stationary exposure measurements

**Fig. 4:**
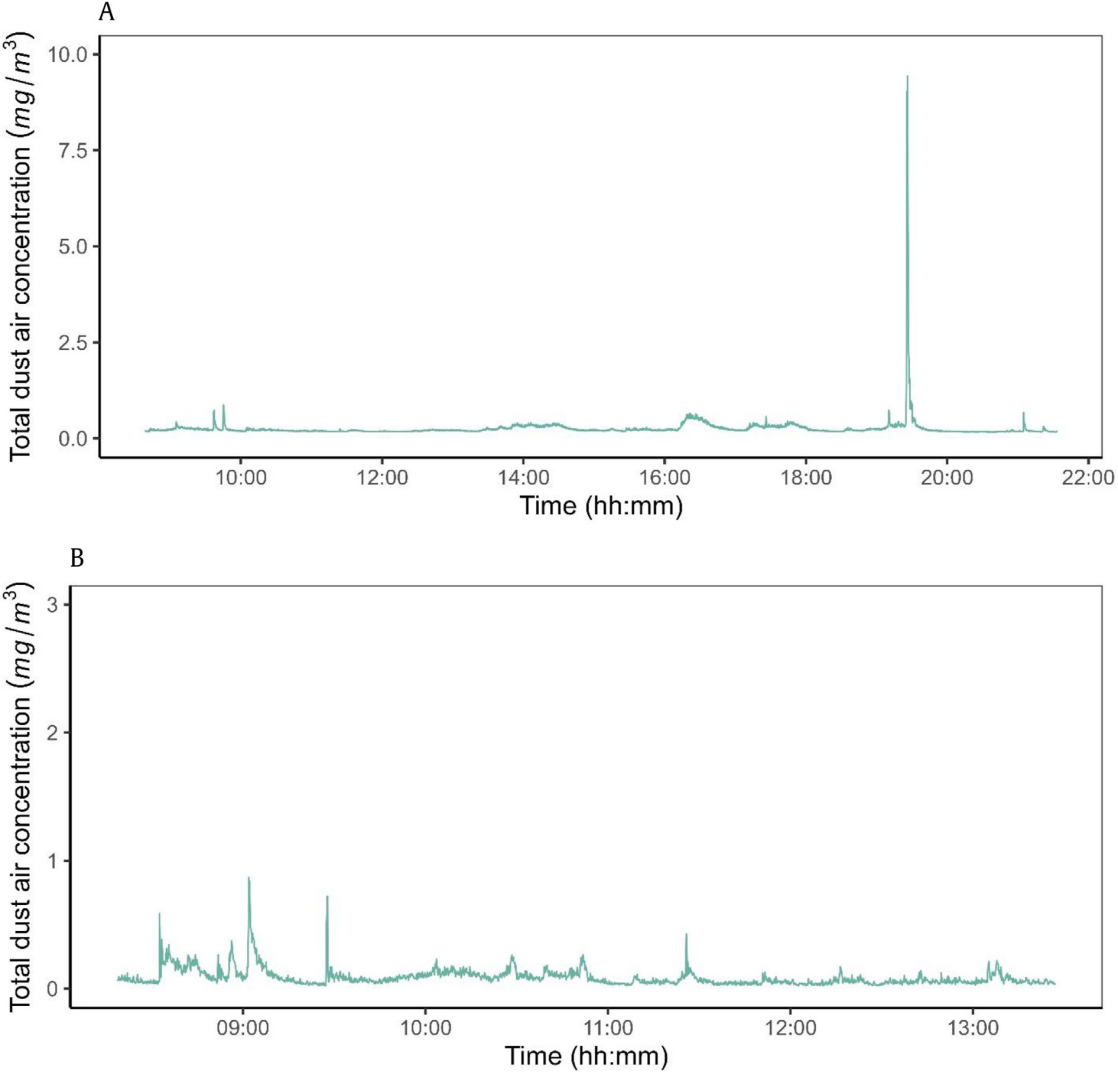
A Stationary direct reading measurements of total dust air concentrations (mg/m^3^) taken every 10 s using a DustTrak DRX aerosol monitor in Department B for two shifts. The *y*-axis shows the total dust concentration in milligrams, while the *x*-axis shows the time in hours and minutes. The results show consistently low total dust concentrations throughout most of the day, but a substantial spike is observed just before 20:00, with total dust concentrations reaching 9 mg/m^3^. B Stationary direct reading measurements of total dust air concentrations (mg/m^3^) taken every 10 s using a DustTrak DRX aerosol monitor during a work shift in Department C. The *y*-axis represents the total dust concentration in milligrams, while the *x*-axis shows the time in hours and minutes. The data reveals a consistently low total dust concentrations for most of the day, with minor fluctuations between 08:30 and 09:20.

Fig. 4A depicts the real-time monitoring results from the DustTrak DRX device, highlighting the air's total dust concentrations in Department B. The graph shows that the total dust concentration remained constant during two shifts, except for a substantial spike at 19:25, contributing to approximately 13% of the day's total dust air concentration as shown in Supplementary S1A (Fig. S1).

In contrast, Fig. 4B shows the total dust air concentration in Department C, covering a single shift. The graph demonstrates that the overall dust concentrations remained steady, with minor fluctuations observed between 08:30 and 09:20. These fluctuations accounted for approximately 23% of the day's total dust concentration, as shown in Supplementary Figure S1B. It is worth noting that the total dust concentration in Department B was 10 times higher than in Department C.

## 4 Discussion

The results of this study show that workers at an instant powder food manufacturing plant may be highly exposed to inhalable aerosols, with a 90th percentile of 10 mg/m^3^ and a maximum of 20 mg/m^3^ (Table 1; Fig. 1). The sampling strategy included all tasks at the plant that involved handling dried materials and powders. Given that the plant produces various products containing allergens, additives, and preservatives, exposure to high aerosol concentrations could pose a risk of adverse health effects for workers, such as occupational rhinitis and asthma.

Table 1 clearly shows that the aerosol concentrations in Department B is considerably higher than those in Departments A and C. This difference can be attributed to the specific tasks performed in Department B, where employees handle powdered substances directly. These tasks involve manual weighing, mixing, and adding various powdered ingredients (such as nutrients, additives, preservatives, and antioxidants) to final products. Manual cleaning methods, such as brushing and vacuuming bulk bags containing products, also contribute to the increased aerosol concentrations in this department. This correlation between manual handling of powders and increased aerosol concentrations is consistent with similar studies conducted in the bakery and grain compound feed industries [2,46]. In addition, Fig. 4 highlights that certain tasks considerably contribute to the overall background concentration of total dust air concentrations observed in Department B. This is supported by studies conducted in bakery environments by Karjalainen et al. and Meijster et al. [47,48], which also found that manual operations lead to peak exposures. In contrast, Departments A and C involve minimal direct contact with powdered materials, focusing primarily on handling big bags through semi-automatic big bag systems during filling and discharge processes. This difference in operational tasks is reflected in the lower aerosol concentrations observed in these departments.

Interestingly, all three departments showed a higher median aerosol concentration in the autumn compared with the summer (Fig. 3). The aerosol concentrations were similar between seasons for Departments B and C, but there was a noticeable difference in Department A. When asked, the factory management stated that the production volume was consistent between the two sampling campaigns. However, the factory produces different products throughout the year, which could generate varying amounts of aerosols and contribute to the observed seasonal differences. The statistically significant difference in aerosol concentrations observed for Department A between summer and autumn can be attributed to the discrepancy in the inhalable aerosol measurements taken during these seasons. Notably, more samples were collected during autumn for workers directly involved in grinding materials into powders than during summer. In the case of Department B, the overall variation in inhalable aerosol concentrations can potentially be explained by individual worker differences. Despite the lower aerosol concentrations observed during summer compared to those during autumn and the relatively similar production volumes between the two seasons, it is reasonable to consider the role of the ventilation system as a potential contributor to the observed seasonal variation in aerosol concentrations.

In contrast, Departments B and C showed relatively stable background air concentrations of total dust for most of the workday (Fig. 4). However, Department B exhibited higher background air concentrations and a tenfold higher accumulated exposure than Department C. This difference in total dust concentrations can be explained by the difference in the manual work tasks described earlier in the respective departments. The increase in dust concentrations may indicate that a specific activity or event within the departments contributes to elevated total dust concentrations. Although these peaks of increased total dust concentrations in Department C were less substantial than the single peak in Department B, their cumulative effect is substantial. These findings highlight the importance of considering not only isolated peaks, but also the frequency and cumulative effect of minor peaks when assessing occupational exposure. Furthermore, this difference highlights the need to recognize variations in aerosol concentrations between different workplace areas, even within the same facility.

The two parallel GSP samplers collected very similar median concentrations of inhalable aerosols, with the left sampler showing slightly higher concentrations (1.1 mg/m^3^ and 1.4 mg/m^3^ respectively, as shown in Fig. 1). The two samplers exhibited a high degree of comparability without any systematic differences (Fig. 2). The observed difference between the two samplers is likely due to the potential inhomogeneity of high aerosol exposure, leading to more noticeable discrepancies between the left and right sides at elevated concentrations. In the food manufacturing industry, where manual handling of ingredients and equipment is common, the choice of hand for specific tasks may result in variations in proximity and duration of exposure to aerosol-emitting sources. Disparities in the spatial distribution of aerosol sources and the orientation of workstations could also contribute to differences in aerosol concentrations based on hand dominance.

Although this study utilized a comprehensive approach, using personal sampling equipment and covering both morning and evening shifts across two seasons, some limitations should be acknowledged. Obtaining detailed information about the factory's ventilation systems proved to be a challenge beyond that presented in the article. To account for seasonal variations, conducting additional measurements throughout the year might be beneficial. Furthermore, the measurements focused solely on the manufactured products during sampling, potentially overlooking other substantial products. Therefore, extending the measurement period over more days and across different seasons could provide a more comprehensive overview. Measuring each worker's specific workload and proportion of time spent on each task was not feasible due to the numerous measurements taken daily across different shifts. In addition, the voluntary nature of participation in the study led to fewer measurements for some operations than initially planned. Maintenance activities during the sampling were also affected the measurements. Despite these challenges, the study successfully included all relevant tasks and departments. [14]

In summary, this study revealed notable discrepancies in aerosol concentrations among workers at a food manufacturing facility, with substantial variation observed between departments and seasons. Employees involved in manual tasks that involve handling powdered ingredients, such as weighing, mixing, and cleaning, were found to have considerably higher concentrations of aerosol exposure. A key finding was the seasonal fluctuation in aerosol concentrations, with autumn showing higher concentrations than summer across all departments. This study highlights the critical need for targeted interventions to address specific tasks that contribute to peak aerosol concentrations, emphasizing the importance of identifying and mitigating high-exposure activities. In addition, the observed differences in aerosol concentrations among individuals and departments, as well as seasons, underscores the necessity for a deeper understanding of exposure dynamics within the food manufacturing process. This study emphasizes the importance of comprehensive exposure assessments in the food industry to develop effective strategies for managing occupational exposure risks.

## Conflicts of interest

The authors declare no conflict of interest relating to the material presented in the article.

## Funding

The study was founded by the National Institute of Occupational Health, Norway.

## Figures and Tables

**figs1:**
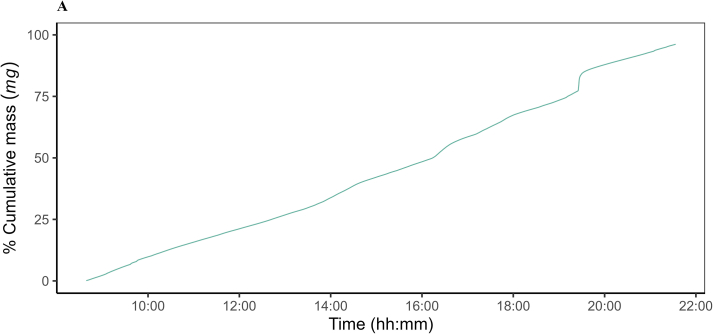
Percentage of the cumulative total dust concentrations measured every 10 s using a DustTrak DRX aerosols monitor during two work shifts in Department B.

**figs2:**
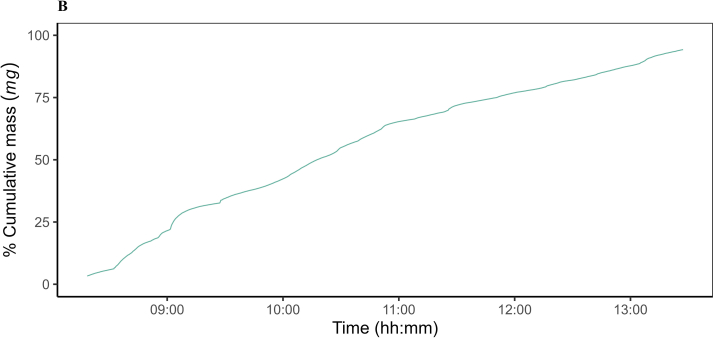
Percentage of the cumulative total dust concentrations measured every 10 s using a DustTrak DRX aerosols monitor during one work shift in Department C .
